# Supplementary material for: Evaluation of Internet-Connected Real-Time Remote Auscultation: An Open-Label Randomized Controlled Pilot Trial
Source: J Pers Med. 2022 Nov 24;12(12):1950. doi: 10.3390/jpm12121950 (PMC9783264; doi:10.3390/jpm12121950)
Supplement: Supplementary file 1 [file jpm-12-01950-s001.zip › jpm-2008749-supplementary.pdf]

Evaluation of Internet-Connected Real-Time Remote Auscultation:
An Open-Label Randomized Controlled Pilot Trial

Day ________________ Sex ________________

Age ________________ Year ________________

group classical auscultation • remote auscultation (4G • Pocket Wifi • LAN)

1. ☐Normal, ☐S3 gallop, ☐Aortic stenosis (AS),
   ☐Aortic regurgitation (AR), ☐Mitral regurgitation (MR)

2. ☐Normal, ☐S3 gallop, ☐Aortic stenosis (AS),
   ☐Aortic regurgitation (AR), ☐Mitral regurgitation (MR)

3. ☐Normal, ☐S3 gallop, ☐Aortic stenosis (AS),
   ☐Aortic regurgitation (AR), ☐Mitral regurgitation (MR)

4. ☐Normal, ☐S3 gallop, ☐Aortic stenosis (AS),
   ☐Aortic regurgitation (AR), ☐Mitral regurgitation (MR)

5. ☐Normal, ☐S3 gallop, ☐Aortic stenosis (AS),
   ☐Aortic regurgitation (AR), ☐Mitral regurgitation (MR)

****

6. ☐Normal, ☐Wheeze, ☐Rhonchi, ☐fine, ☐coarse

7. ☐Normal, ☐Wheeze, ☐Rhonchi, ☐fine, ☐coarse

8. ☐Normal, ☐Wheeze, ☐Rhonchi, ☐fine, ☐coarse

9. ☐Normal, ☐Wheeze, ☐Rhonchi, ☐fine, ☐coarse

10. ☐Normal, ☐Wheeze, ☐Rhonchi, ☐fine, ☐coarse

Figure S1. Questionnaire format for the identification of the cardiopulmonary sounds.
